# Habitat Selection and Reproductive Success of Lewis's Woodpecker (*Melanerpes lewis*) at Its Northern Limit

**DOI:** 10.1371/journal.pone.0044346

**Published:** 2012-09-18

**Authors:** Xiang Zhu, Diane S. Srivastava, James N. M. Smith, Kathy Martin

**Affiliations:** 1 Department of Zoology, University of British Columbia, Vancouver, British Columbia, Canada; 2 Centre for Applied Conservation Biology, University of British Columbia, Vancouver, British Columbia, Canada; University of Utah, United States of America

## Abstract

Lewis's Woodpecker (*Melanerpes lewis*) has experienced population declines in both Canada and the United States and in 2010 was assigned a national listing of threatened in Canada. We conducted a two-year study (2004–2005) of this species at its northern range limit, the South Okanagan Valley in British Columbia, Canada. Our main objective was to determine whether the habitat features that influenced nest-site selection also predicted nest success, or whether other factors (e.g. cavity dimensions, clutch initiation date or time of season) were more important. Nest tree decay class, density of suitable cavities and total basal area of large trees were the best predictors of nest-site selection, but these factors were unrelated to nesting success. Estimates of demographic parameters (mean ± SE) included daily nest survival rate (0.988±0.003, years combined), nest success (0.52±0.08), clutch size (5.00±0.14 eggs), female fledglings per successful nest (1.31±0.11), and annual productivity (0.68±0.12 female fledglings per nest per year). Although higher nest survival was associated with both early and late initiated clutches, early-initiated clutches allowed birds to gain the highest annual productivity as early clutches were larger. Nests in deep cavities with small entrances experienced lower predation risk especially during the peak period of nest predation. We concluded that nest-site selection can be predicted by a number of easily measured habitat variables, whereas nest success depended on complicated ecological interactions among nest predators, breeding behaviors, and cavity features. Thus, habitat-based conservation strategies should also consider ecological factors that may not be well predicted by habitat.

## Introduction

Understanding habitat selection and its influence on reproductive success is crucial to species recovery planning. Habitat protection has proven one of the most effective measures to rescue and recover threatened and endangered species [1]. However, successful implementation of management plans depends upon our knowledge of habitat requirements of target species [2]. By habitat we mean the vegetative, structural, edaphic and topographic features of an area, measured at scales relevant to the daily needs of the organism (e.g. resource extraction, territoriality or reproduction). Habitat is usually considered something that an organism selects, not constructs (thus ponds are beaver habitat, whereas dams are part of the beaver's extended phenotype). Habitat requirements of species are often estimated as habitat where the species occurs, with the logic being that a species will select habitat where it obtains maximum fitness. For example, several studies have shown that habitat features affect foraging conditions of breeding birds and risk of nest predation, both important determinants of reproductive success [3], [4], [5]. However, habitat selection may not be a reliable index of habitat-specific fitness for several reasons. First, individuals may be attracted to a poor habitat (an ecological “trap”) by misleading cues [6], [7]. Recent changes in the environment caused by exotic species can cause nest-site choices of birds to become maladaptive [8], [9]. Second, some components of fitness may be primarily determined by ecological factors unrelated to the habitat, such as timing of reproduction or intensity of nest predation [10], [11]. Finally, habitat selection may be constrained by ecological factors such as interspecific competition, such that individuals are rarely able to use the habitat that would otherwise maximize fitness [3],[5],[11].

Lewis's Woodpecker populations have been declining over the last 40 years because of the loss of suitable habitat due to fire suppression, forest cutting, agricultural development, water management practices and urbanization [12], [13], [14]. Since the 1970s, the range of Lewis's Woodpecker in Canada, restricted to southern British Columbia, has contracted east from southeastern Vancouver Island and the lower Fraser Valley to the southern interior of B.C. with extirpation of the coastal sub-population. In the southern interior of B.C. the species now breeds only in the area from the Similkameen Valley east to the East Kootenay Trench, with highest abundance centered in the Okanagan Valley [12], [15], [16]. In 2001, Lewis's Woodpecker was listed as a species of special concern in Canada, and a recent re-assessment listed the species as threatened [17]. In order to develop a recovery plan, understanding habitat selection and reproductive success of Lewis's Woodpeckers is vital.

Previous studies on Lewis's Woodpeckers have examined either nest-site selection or effects of habitat on reproductive success. Nest-site selection appears to be influenced by features both of the landscape [e.g. vegetation type and fire history; 18], [19] and of individual trees [e.g. nest cavity position and tree condition; 20], [21], [22]. Nest success is also influenced by vegetation type and fire history, as well as attributes of the nest tree [14], [19], [23]. However, despite this extensive research on the reproductive biology of Lewis's Woodpecker, only one study to date has yet examined whether this species select nest-sites based on features that optimize nest success: Newlon and Saab [19] recently showed that in aspen woodland in Idaho, habitat features correlated with nest-site selection were poor predictors of nest success. The current study examines this question in a very different habitat and geographical region, and compares precisely the same habitat models for nest site selection and nest success.

Our objectives are two-fold. First, we examine which habitat features determine nest-site selection by this species near its northern range limit. Previous studies of habitat and nest-site selection by Lewis's Woodpecker are centered on the western U.S. [e.g. 18], [20], [21], [22] and may not apply to the less well-studied populations at the northern range limit [12]. Such peripheral populations may become vital as species adapt to directional changes in climate or diminished habitat quality. Second, we ask how important the preferred habitat features are in determining nest success. Several outcomes are possible here: (1) Habitat features used in nest-site selection optimize nest success. In this case, these habitat features are a useful guide for developing a conservation strategy for this species. (2) Habitat features used in nest-site selection lead to reduced nest success. This would suggest an “ecological trap”, whereby managing for these habitat features may create or perpetuate sink population dynamics. (3) Habitat features used in nest-site selection may lead to higher nest success, but either nest success or nest-site selection is constrained by ecological factors unrelated to these habitat features, such as timing of nest initiation, predation or interspecific competition. In this case, habitat features are useful for conservation, but only in the context of a broader strategy that also considers other factors. (4) Habitat features used in nest-site selection are unimportant relative to other ecological factors in determining nest success. In this case, habitat-based conservation strategies should pay particular attention to these ecological factors.

## Methods

### Study species

Lewis's Woodpecker, a short distance migratory bird, tends to form long-term pair bonds [24]. Pairs produce one brood annually and may re-nest if the first attempt fails. Both sexes participate in incubating and nestling care, which lasts for 13–15 days and 28–34 days respectively. The entire breeding cycle, plus laying period, lasts for 52–58 days [24]. In British Columbia, clutch sizes generally range from 4–6 eggs [25]. Lewis's Woodpecker is a weak excavator; it often nests in existing cavities initially excavated by other woodpeckers such as the Northern Flicker (*Colaptes auratus*), and tends to return to the same nest-sites in subsequent years [24]. In the breeding season, the bird is mainly an aerial insectivore with fruit as a secondary part of its diet depending on local availability [24], [26]. Lewis's Woodpecker prefers open ponderosa pine (*Pinus ponderosa*) forest, riparian woodland dominated by black cottonwood (*Populus balsamifera trichocarpa*) or aspen (*Populus tremuloides*) and partially logged or burned pine forest [19], [24], [27]. These habitats, which used to be maintained by periodic wildfires, provide the species with abundant nesting sites and good visibility for catching insects in the air [24], [28], [29], [30].

### Study area and nest surveys

The study, conducted during 2004–2005 in the South Okanagan Valley of interior BC, Canada (W119°20′–119°45′, N49°–49°30′), included six areas ranging in elevation from 320–1100 m, each with one to nine pairs of Lewis's Woodpeckers/site/year: Chopaka Grassland Protected Area (∼500 ha), Spotted Lake Grassland Protected Area (∼430 ha), Vaseux Lake Ecological Reserve (∼2400 ha), Kilpoola Grassland Protected Area (∼800 ha); Sun Oka Provincial Park (40 ha) and surrounding areas (∼1500 ha). All sites were dominated by ponderosa pine (*Pinus ponderosa*), but also included semi-arid grassland, shrub steppe, black cottonwood open forest, mixed coniferous and broadleaved open woodland, and Douglas-fir (*Pseudotsuga menziesii*) forest (Table S1). Because the study areas are all located in the same eco-region and overlapped broadly in habitat conditions, we pooled all areas to maximize sample size. To account for any spatial autocorrelation in habitat we incorporated study area as a random factor in analyses of factors for nest selection or success.

From early May to late August, we conducted four rounds of intensive surveys to search for nests and adults, with consistent effort among all the study areas and years. Once a nest was found, we visited it at 3–4 day intervals until either fledging or failure. During each nest visit, we first observed adult breeding behavior for one hour (time spent on roosting, courtship, copulation, incubating, provisioning, alarm calling, and cavity and territory defense). When feasible, we accessed the nest using a climbing rope and documented the contents of the nest with a Sony DSC-U20 digital camera inserted into the nest hole. We were able to estimate nest age for most nests through comparisons of digital photographs taken over time, plus behavioral observations of parent birds. We defined nest age as time (in days) from the laying of the first egg. We considered a nest successful if at least one young fledged. When it was not feasible to access the nest cavity, we used our observation data to assign nesting status (active, feeding young, terminated, etc).

### Selection of explanatory variables

We anticipated that nest-site selection and nest survival would be based on habitat features linked to cavity availability and predation risk. Because weak cavity excavators like Lewis's Woodpeckers mainly use pre-existing cavities [23] or excavate cavities in trees with advanced decay, they may be limited by the density of suitable cavities and the appropriate decay class of trees [31]. This species is reported to prefer old-growth habitat [13], and the degree to which habitat approached this state was quantified as the basal area of large (diameter at breast height [DBH] ≥50 cm) trees per hectare. Lewis's Woodpeckers are reported to prefer open habitats with dense shrubs [28], , so we included measures of vegetation type (as described shortly). Elevation can affect the start of the growing season, frequency of inclement weather, and availability of food resources [13]. We intentionally included in our study habitat variables varying from the scale of individual trees (nest tree decay class) to the 50 m around nests (vegetation cover and cavity density) to landscapes (elevation), as there is evidence that nest-site selection in Lewis's Woodpeckers is affected by processes operating at multiple scales [e.g. 21].

In addition to these habitat features, we also considered cavity features and time of season. The degree to which cavity-nesting birds evade nest predators depends on cavity features such as cavity shape or height above ground [31]. Deep cavities with small entrances may protect against predators such as squirrels, weasels (*Mustela spp.*), and snakes [12], [21], [33]. Cavity height may affect vulnerability to ground-dwelling mammalian predators [34].Seasonal patterns in daily nest survival rate are reported for Lewis's woodpeckers [19] and other bird species [35], [36], [37], and there may be complex interactions between the time of season and stage in the nesting cycle [38]. We characterized these temporal trends as time of season, nest age, clutch initiation date and year.

### Habitat surveys

We conducted vegetation surveys at 50 nesting sites (involved 57 nesting attempts over both years) and 28 random sites after the breeding season (Table S1). We sampled random sites to compare habitat surrounding nests with habitat available in the landscape; time constraints precluded collecting data from more random sites. We located random sites within each study area using randomly generated coordinates. In each site, we centered a 50 m-radius plot (0.79 ha) on the nest tree or a randomly selected tree with DBH≥50 cm (minimum used by Lewis's Woodpeckers in this study) for tree measurements. If there was no suitable tree in the random site, we moved the plot centre to the nearest suitable tree. None of the central trees in our random sites contained Lewis woodpecker nests. For shrub measurements, we positioned five 10 m-radius subplots at the 50 m end of the radius in each of the four cardinal directions, and at the center of the plot. At the center of each subplot, we added a 5 m-radius plot for grass and forb measurements. Since the subplots covered the extent of the 50 m plot, the average of grass or shrub cover in the subplots provide an estimate of the average cover for the entire 50 m plot, and thus provides habitat information on the primary foraging areas closest to the nest for each pair. We justify this approach by noting that habitat associations documented for Lewis's Woodpeckers at scales as small as 0.04 ha are congruent with those at the landscape scale [18].

In each site, we measured seven habitat variables. In the 50 m-radius plots we recorded elevation (m above sea level) at the central tree, live tree canopy cover (% cover visually estimated), density of suitable cavities (total number of suitable cavities per ha, with suitability assessed by visual estimation of the entrance size), and basal area of large trees per ha (total cross-section area of trees at 1.3 m height with DBH≥50 cm). We visually estimated shrub cover (%) and grass cover (%) in the 10 m and 5 m-radius plots, respectively. We averaged grass and shrub cover in the subplots to provide an estimate of the average cover for each in the entire 50 m plot. Finally, we recorded decay class of the central tree as 1 = live tree, 2 = dead-top tree, 3 = dead tree with all branches present, 4 = rotten dead tree with >50% branches missing [modified after 39].

### Cavity features

For all nest trees, we measured the following features of the cavity (virtually no centre trees in random plots contained cavities): height (m) of the nest cavity above the ground measured with a clinometer and the ratio of vertical hole depth to entrance size (a measure of cavity quality; entrance size was estimated as the product of hole width and hole height, all ±1 mm). We used a plumb line to measure the vertical cavity depth from the bottom of the cavity to the lower edge of the entrance. We transformed this ratio into a unitless value >0.01 by multiplying by 100 mm.

## Statistical Analyses

### Overall reproductive parameters

We estimated daily nest survival rate, nest success, clutch size, female fledglings per successful nest, and annual productivity for the entire study period using all nests. We estimated daily nest survival rate using a null model (i.e. a model fitted with just a constant) of Mayfield logistic regression [37]. Because there was no significant year effect on daily nest survival rate (p = 0.61), we pooled the two years of data. We calculated nest success by raising daily nest survival rate to the 54th power (the average number of days for a complete nesting cycle, see below). We determined annual productivity, which is the number of female fledglings per nest per year, by taking the product of nest success and the average female fledglings per successful nest. We calculated the standard error of annual productivity using the moment estimator of a product of independent variables [40].

### Nest-site selection

We used mixed logistic regression models to test how the habitat around a tree, and created by the tree, affects the probability of that tree being selected for nesting by Lewis's Woodpecker [41]. We did not consider cavity features in this model, for two reasons. First, Lewis's woodpeckers will sometimes excavate their own cavities in decayed trees, so cavity characteristics cannot be assumed to be independent of the presence of a Lewis's woodpecker. Second, there were virtually no cavities in the centre trees in our random plots, so in practical terms there is no null distribution of cavity features with which to compare the utilized cavities. However, our model does include nest/centre tree decay class and the density of suitable cavities, which together cover both aspects of Lewis's woodpeckers nesting strategy: the adoption of existing cavities or the creation of new cavities in decayed trees. Our global model included all seven habitat variables, with study area as a random effect. We compared this global model with 23 candidate models and a null model using Akaike information criterion with correction for small sample size [AICc, 42].

Rather than test all possible subsets of the seven habitat variables, we used an information-theoretic approach whereby we constructed candidate models which seemed most probable based on previous work, most parsimonious given the system, and most biologically meaningful. Previous work on woodpeckers has shown that the most important determinant of population density is often the number of pre-existing cavities or trees suitable for cavity excavation [43], [44], [45]. We therefore constructed our candidate models such that most contained the density of suitable cavities and/or nest tree decay class, the two habitat variables most directly related to the potential availability of cavities; we consider these core variables. The variables elevation, stand maturity and relative ratio of trees to other vegetation types formed a second tier of variables incorporated in models after the core variables. The final two variables, shrub and grass cover, were evaluated after other variables had been incorporated in the model, as Lewis' Woodpeckers prefer open areas with dense shrubs. We also included as candidate models each habitat variable by itself to ensure that the most parsimonious model was chosen. We did not include interactions between habitat variables, as such interactions were not found in other studies, and in any case the biological meaning of such interactions is unclear. We examined only linear effects, as many habitat variables encompassed a small range of values (quadratic effects are only revealed with a broad range in values), and we did not want to overfit our model. Although some habitat variables covaried with each other (correlation coefficients from −0.53 to +0.57; Table S2), these correlations were not strong enough (i.e. absolute value of coefficient was always less than 0.8) to substantially affect model selection.

### Nest survival and nest success

We used mixed Mayfield logistic regression to investigate impacts of habitat features, cavity features, and temporal factors on daily nest survival rate [46], [47]. We coded the dependent variable, daily nest survival, as “1” for each day a nest continued to survive and “0” for the day when the nest failed. As this variable is binomial, we used a logit link function. Specifically, the daily nest survival rate (Si) took the formulation of:
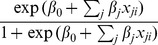
. *β_0_* represents the intercept; *β_j_* represents the *j*th parameter; *x*_ji_ denotes the *i*th observation of the *j*th covariate. *β_0_* and *β_j_* are parameters to be estimated. [46] pointed out that this formulation allows daily nest survival rate to vary among groups of nests (i.e., group specific covariates), among individual nests (i.e., nest specific covariates), and among days (i.e., time specific covariates). Given anticipated correlations between different observations on the same nest, or between different nests in the same study area, we incorporated both study area and nest identity as random effects in the mixed models.

We outlined two competing general hypotheses in the form of global models: (1) the same habitat variables found to be important in nest-site selection are also important for daily nest survival rate, (2) cavity features and temporal factors determine daily nest survival rate. We first separately assessed the importance of habitat variables and temporal/cavity models, and then compared simultaneously the two global models and all associated candidate models. For the habitat variables, we used an identical global model and associated candidate models to those used for nest-site selection. The other global model included cavity features (cavity height, ratio of hole depth to entrance size) and temporal factors (year, time of season, nest age, and clutch initiation date). Here we again used the literature to identify two core variables - nest age and clutch initiation date - that are consistently shown to be correlated with daily nest survival in other species [35], [36], [37], and recently in Lewis's woodpeckers [19]. Multiple regression models always contained one or both core variables. We also investigated the additional effects of cavity height and cavity entrance∶depth ratio, clutch initiation date calculated as the number of days since May 10, and year. Because nest failures often occurred at intermediate nest ages, we included both linear and quadratic effects of time of season, nest age, and clutch initiation date.

For analysis of daily nest survival rate, we assumed that if a nest failed between two nest visits, the failure occurred in the midpoint of the interval. Previous work [48] has concluded that this assumption is justifiable when the interval is short (<one week) and daily nest survival rate is high (>0.90), both conditions typify our study. To avoid psuedoreplication, if a cavity was used in both years, only the 2004 data were included for the analysis of the effects of habitat features on nest-site selection and daily nest survival rate. We also eliminated five nests for which we lacked accurate information about nest age or fate required by the Mayfield logistic regression method [37]. Thus, we used 45 nests for regression analysis. Stata 10.0 [49] was used for all analyses.

Because nest survival over each day is assumed to be independent between days in Mayfield logistic regression, we can estimate nest success as a consecutive accumulative product of daily nest survival rate from nest age one through to fledgling. In our study, daily nest survival rate (Si) was modeled as a function of habitat and cavity features (X), nest age (NA) and clutch initiation date (C). Thus, nest success can be estimated as:
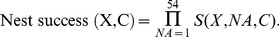
(1)


We used a nest age of 54 days to estimate nest success (54.22 days ±0.75, *n* = 18 nests followed from first egg to fledge date). This nesting cycle was composed of a 6–9 day laying period, a 14–15 day incubation period, and a 30–34 day nestling period.

### Annual productivity

Annual productivity depends on (a) the probability of nest success (estimated by Eq. 1), (b) the average number of fledglings produced from a successful nest, and (c) the proportion of fledglings that are female. There is no published information on the sex ratio in Lewis's woodpecker, nor were we able to directly determine the fledgling sex ratio as we intentionally did not capture individuals of this threatened species. However, the sex ratio in the congeneric *Melanerpes formicivorus* (acorn woodpecker) has been exhaustively studied, and does not differ significantly from 1∶1 at the hatching stage, although it starts to be very slightly male-biased (54∶46) by fledging [50]. We conservatively assumed a 1∶1 fledging sex ratio for Lewis's woodpecker, as have other authors [14], as the slight female bias in pre-fledging mortality in *M. formicivorus* may be related to cooperative breeding [50], and so not apply to Lewis's woodpecker. To calculate the dependence of (b) on time, we used ordinal logistic regression to model the relationship between clutch initiation date and the number of fledglings produced per successful nest. Ordinal logistic regression estimates the likelihood of producing 1, 2, 3, 4 or 5 fledglings (the maximum number of fledglings observed was 5). So, combining (b) and (c), the expected number of female fledglings per successful nest was:
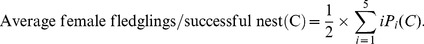
(2)


Here, *P_i_* (C) represents the probability to produce *i* fledglings as a function of clutch initiation date (C). Multiplying Eq. 2 with Eq. 1, the annual productivity of Lewis's Woodpeckers was estimated as:
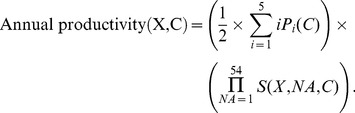
(3)


Here, X denotes a series of covariates. We assigned 10 May (the earliest clutch initiation date in our study) as day one.

All data are presented as means ± SE unless otherwise specified and the significance level for all tests was set at α<0.05. Statistical analysis was performed using Stata version 10.1 (StataCorp 2009).

## Results

In total, we found 57 nests, comprising 36 nests in 2005 and 21 in 2004 (Table S1). Overall, nest success was 0.52 (Table 1), with estimates varying from 0.12 to 0.77 between study areas (Table S1). Of the successful nests, an average of 2.6 fleglings per nest were produced. Assuming a 1∶1 sex ratio, we therefore estimate 1.3 female fledglings per successful nest (Table 1). Of the failed nests, 15 (75%) were depredated, three (15%) were deserted, and two (10%) nests were destroyed by natural elements (one nest tree was blown down, one cavity filled with rainwater drowning the nestlings).

**Table 1:** Overall reproductive parameters of Lewis's Woodpeckers in the South Okanagan Valley, British Columbia, 2004–2005. Annual productivity was the product of nest success and female fledglings per successful nest.

Overall reproductive parameters	Mean	95% CI lower	95%CI upper	Nests
Daily nest survival rate	0.988	0.982	0.994	45
Nest success	0.52	0.36	0.68	45
Clutch size	5.00	4.73	5.27	31
Female fledglings/successful nest	1.31	1.09	1.53	29
Annual productivity	0.68	0.44	0.92	-

**Figure 1:**
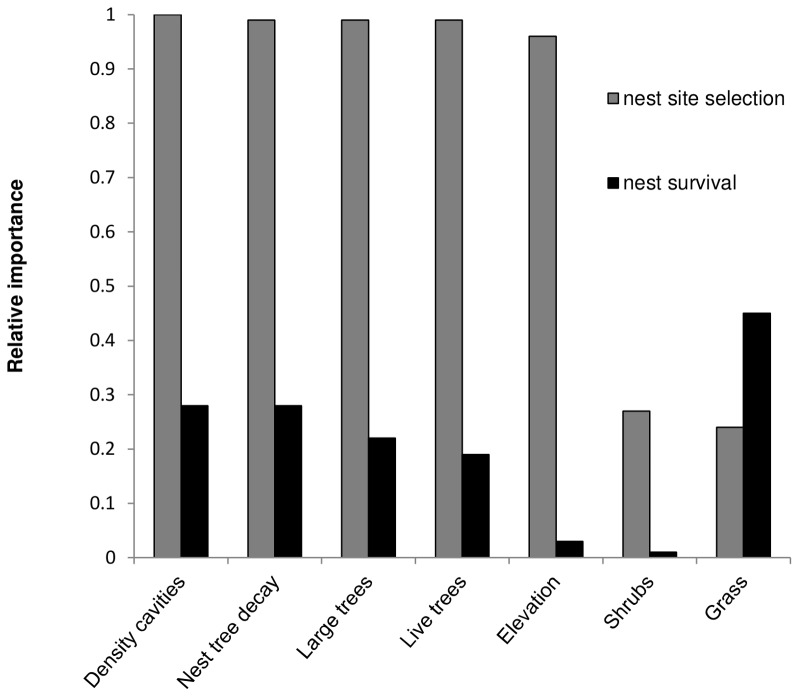
Relative importance of habitat variables in predicting either nest site selection or nest success. Relative importance is calculated as sum of weightings for a variable in all models where this variable was included, and is constrained between 0 and 1.

Sites with nests differed significantly from sites centered on randomly-selected trees in most habitat variables (Table 2). In the best model explaining nest-site selection (lowest AICc in models summarized in Table 3 and in full in Table S3 ), nest sites were most likely to be selected where there were more suitable cavities (relative importance of 0.99, calculated as sum of weightings for a variable in all models where this variable was included; Fig. 1), a more advanced nest tree decay class (relative importance of 0.99), greater total basal area of large trees (0.96), higher live tree canopy cover (0.93), and lower elevation (0.96). Note that the 95% C.I. for the last two parameters overlapped zero (Table 3) and thus these habitat features contribute to the ability of the overall model to explain variance but do not in themselves predict nest-site selection.

**Table 2:** Habitat and cavity features (mean ± SE) of nest sites and random sites and of successful and failed Lewis's Woodpecker nests in the South Okanagan Valley, British Columbia, 2004–2005. All 45 nest sites were actively used by Lewis' Woodpeckers, and the fate of nests in nest sites was subsequently determined to be either successful or failed. Differences between nest and random sites, and between successful and failed nests, assessed with t-tests.

Habitat and cavity features	Code	Nest sites (n = 45)	Random sites (n = 28)	P	Successful nests (n = 29)	Failed nests (n = 16)	P
Elevation (m)	EL	688±39	737±34	0.39	663±52	733±58	0.40
Live tree canopy cover (%)	TC	6.63±0.81	14.17±2.92	0.00	6.50±0.98	6.87±1.45	0.83
Shrub cover (%)	SC	14.90±1.21	18.54±2.34	0.13	15.08±1.40	14.58±2.35	0.85
Grass cover (%)	GC	42.07±1.66	34.30±2.34	0.01	44.55±1.70	37.57±3.31	0.04
Basal area of large trees (m^2^ per ha)	BA	3.32±0.38	2.04±0.33	0.02	3.32±0.50	3.32±0.59	1.00
Nest tree decay class	ND	2.89±0.14	1.93±0.22	0.00	2.79±0.17	3.06±0.25	0.37
Density of suitable cavities (per ha)	DS	4.73±0.47	0.54±0.33	0.00	4.92±0.67	4.38±0.58	0.59
Cavity height (m)*	CH	10.71±0.72	-	-	11.19±0.78	9.84±1.44	0.37
Ratio of vertical hole depth to entrance size* a	DE	0.67±0.02	-	-	0.70±0.03	0.62±0.03	0.10

* Only applicable to nesting sites.

a Ratio transformed into a unitless index >0.01 by multiplying by100 mm.

**Table 3:** Top three models of all habitat-based models that predict Lewis's Woodpecker's nest-site selection (*n* = 73). Please see Table S3 for the other 21 lower ranking models.

Model	K	−2log(L)	AICc	ΔAICc	Wi
EL+TC+BA+ND+DS	6	30.23	43.50	0.00	0.49
EL+TC+BA+ND+DS+SC	7	29.59	45.31	1.81	0.20
EL+TC+BA+ND+DS+GC	7	30.20	45.92	2.42	0.14

K = number of parameters in the model; −2log(L) = maximum likelihood of the model using natural logarithms; AIC_c_ = Akaike's Information Criterion for small samples; ΔAIC_c_ = adjusted AIC_c_ relative to the top model; W_i_ = AIC_c_ weight; habitat codes given in Table 2. The logit equation (with 95% CI for each parameter in parenthesis) for the best model of nest-site selection (NSS) was: Logit (NSS) = 2.02 (−2.85, 6.88)−0.012 (−0.021,−0.002) * EL−0.14 (−0.28, 0.0003) * TC+0.60 (−0.16, 1.22) * BA+1.72 (0.29, 3.15) * ND+0.93 (0.35, 1.51) * DS.

Successful and failed nests did not differ in most habitat attributes, save grass cover (Table 2). Further, the habitat features found to be most important in nest-site selection proved to be poor predictors of daily nest survival rate (Fig. 1,Table S5). Percentage grass cover was the only habitat variable that was slightly better than a null model (ΔAICc = 2.16) in predictive ability, and it had a low importance value (0.60). All excluded habitat variables also had low importance in predicting nest survival (Fig. 1), including percentage live tree canopy cover, total basal area of large trees, nest tree decay class, and density of suitable cavities (range of 0.25 to 0.38), and percentage shrub cover (0.23) and elevation (0.24).

**Figure 2:**
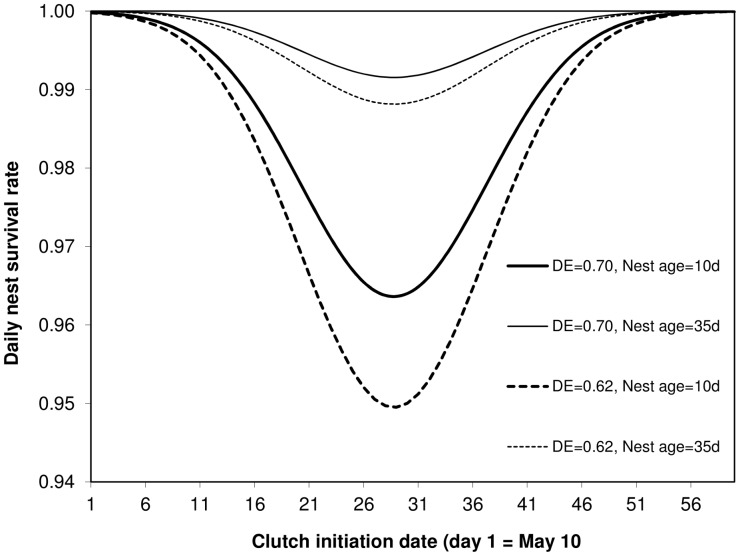
Daily nest survival rate is predicted to be minimal for clutches initiated mid-season, soon after initation (nest age = 10 d vs. 35 days) and in cavities with a low ratio of hole depth to entrance size (DE). Solid and broken curves are indicated for DE = 0.70 (average for successful nests) and DE = 0.62 (average for failed nests), respectively.

When we compared nest survival models containing only habitat variables with the models containing cavity/temporal factors within the same AICc ranking framework, the latter were always ranked at the top, whereas the former was ranked at the bottom (Table S6). In fact, the best habitat model for nest-site selection was, for nest survival, the 40^th^ worst model out of all 41 models examined, more than 22 AICc units worse than the best cavity/temporal model, and substantially poorer fit than even the null model (by 5.5 AICc units). The best model for nest survival included quadratic effects of clutch initiation date and nest age, and linear effects of cavity depth∶ entrance ratio (summarized in Table 4 and in full in Table S4). This model predicted that the lowest daily nest survival rate was experienced by birds that begin laying around 10 June, by nests around 18 days of nest age (mid to late incubation) and in cavities with high ratios of hole depth to entrance size (Fig. 2).

**Table 4:** Top three models based on cavity and time that predict Lewis's Woodpecker's daily nest survival, *n* = 1251 total intervals within 45 nests.

Model	K	−2log(L)	AICc	ΔAICc	Wi
DE+C+C^2^+NA+NA^2^	6	144.35	156.42	0.00	0.42
CH+C+C^2^+NA+NA^2^	6	146.18	158.25	1.83	0.17
DE+NA+NA^2^	4	150.69	158.72	2.30	0.13

Please see Table S4 for the other 14 lower ranking models. Abbreviations as in Table 2 and 3, and in addition: C = clutch initiation date; NA = nest age; T = time of season. The logit equation (with 95% CI given in parenthesis after each parameter) for the best model of daily nest survival rate (DSR) was: Logit (DSR) = 7.90 (1.97, 13.80)+4.28 (0.12, 8.45) * DE−0.41 (−0.79, −0.03) * CI+0.0071 (0.0002, 0.0140) * CI^2^−0.24 (−0.47, −0.01) * NA+0.007 (0.001, 0. 013) * NA^2^.

**Figure 3:**
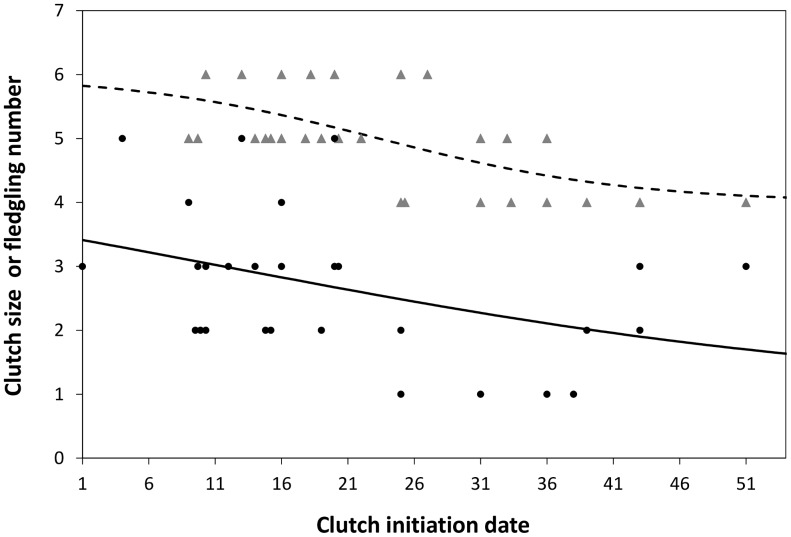
Seasonal patterns of clutch size (grey triangles, dashed line) and fledglings per successful nest (solid circles, solid line) in relation to clutch initiation date. Lines indicate the predicted average values from logistic regressions.

**Figure 4:**
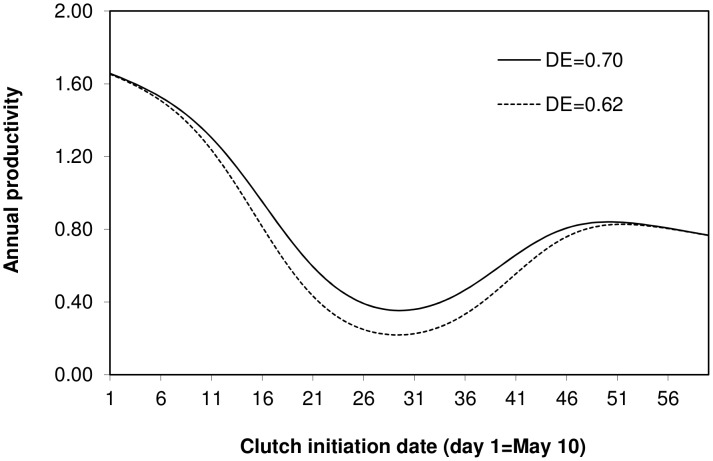
The predicted relationships between clutch initiation date and annual productivity for two cavity shapes (DE = ratio of cavity depth to entrance size). Solid and broken curves indicated when DE = 0.70 (average for successful nests) and DE = 0.62 (average for failed nests), respectively.

Clutch size and fledglings per successful nest varied with clutch initiation date (Fig. 3). If birds started laying late, there was a substantial reduction in the probability of producing a large clutch (*r* ^2^ = 0.29, *P* = 0.001, *n* = 30 nests), and this resulted in fewer female fledglings per successful nest (*r* ^2^ = 0.13, *P* = 0.02, *n* = 28 nests). No habitat variables except elevation influenced clutch size or fledglings per successful nest: Lewis's Woodpeckers nesting at higher elevations started laying later (*r* ^2^ = 0.14, *P* = 0.01, *n* = 45 nests), and thus had smaller clutches (*r* ^2^ = 0.16, *P* = 0.03, *n* = 45 nests). Annual productivity was greatest for nests initiated mid-season in deep cavities with small entrances (Fig. 4).

## Discussion

The basic tenet of habitat selection theory is that species select habitats that optimize their fitness. However, in the case of Lewis's Woodpecker in southern British Columbia, the habitat factors that predicted nest-site selection were poor predictors of nest success. Before examining potential explanations for this apparent paradox, we first examine why Lewis's Woodpeckers selected certain habitats for nests, and what determined nest success.

### Which habitat features predict nest-site selection?

In the South Okanagan Valley, we found that Lewis's Woodpeckers preferentially chose decayed nest trees surrounded by trees with suitable cavities. As in [51], [52], but contrary to [21], we found nest tree decay class but not cavity height to be important. Lewis's Woodpecker nests were reported to be common in sites with standing dead and decaying live trees that range in density from 2.5 trees/ha in Oregon and Washington [53] to ca. 220 dead and decaying trees/ha in Idaho [54]. These habitat characteristics may reflect the particular traits of Lewis's Woodpeckers. Decayed trees with heart rot are important habitat components for most cavity-nesting species, especially weak excavators such as Lewis's Woodpeckers, Downy Woodpeckers (*Picoides pubescens*), and nuthatches [Sitta sp., 31], [52]. For Lewis's Woodpeckers, advanced decay status can make cavity excavation easier, provide nest-lining materials, allow food caching, and increase drumming volume. The exception may be aspen forest in Idaho, where abundant cavities in live trees result in more Lewis's woodpecker nests in live than dead trees [19].

As found in our study, Lewis's Woodpeckers in Colorado, Wyoming, and California avoid dense forests for breeding, favoring open habitat with low ground cover [22], [32], [51], [52]. Nest sites were found in recently burned areas as well as old growth riparian black cottonwood and mixed coniferous-broadleaved woodlands. Our findings largely agree with previous studies in the western United States that Lewis's Woodpeckers prefer recently burned sites [18], [20], [22], [52], [54], and inhabit both cottonwood and ponderosa pine forests [18], [21], [51], [54].

### What determines nest success?

In this study, nest success of Lewis's Woodpeckers was primarily determined by clutch initiation date, nest age and cavity shape. Clutches initiated both early and late in the breeding season had higher daily survival than those initiated in mid-June. Nest survival was lowest 18 days after initiation (mid to late incubation). It is possible that both temporal patterns are related to changes in predation risk as generalist nest predators such as red squirrels (*Tamiasciurus hudsonicus*) may switch to searching for cavity nests when they are most plentiful (mid-season) after which other foods may become available. However it is also possible that the factors responsible for the variation in initiation date also determined temporal variation in nest survival. A number of factors may be responsible for the variation in clutch initiation date, including characteristics of the individual birds (age, quality or competitive ability), annual variation, and habitat characteristics. For example, we found that birds at higher elevation initiated clutches later. It is not possible to evaluate bird quality or annual variability without a much longer-term study involving marked individuals. Only one other study on Lewis's woodpecker has considered within-season temporal patterns, and found that daily nest survival decreased monotonically with initiation date and increased with daily maximum temperature[19], contrasting with the parabolic effect of clutch initiation date on daily nest survival that we documented. In that case, the authors speculated that the effects of initiation date reflected bird quality or synchronization with food availability [19]. Studies on other bird species have also found strong non-linear effects of clutch initiation date and nest age [10], [35], [55].

The most successful nests occurred in cavities with small entrances and deep holes. Nest predation was the major cause of nest failure in Lewis's Woodpeckers, and deep cavities with small entrances may be the best shape to exclude access by predators such as weasels, red squirrels and northern flying squirrels (*Glaucomys sabrinus*) [31]. Some studies in a sub-tropical cavity-nesting community, also found a strong preference for deep cavities with small entrances [56]. This may be a general preference among cavity-nesting birds.

Combining seasonal patterns in nest success and fledglings per successful nest, we found that early clutch initiation allowed Lewis's Woodpeckers to gain the highest annual productivity, corroborating similar effects of clutch initiation date for this species in Idaho [19] and following the general trend for birds that early initiating individuals have higher annual breeding success [e.g. 57]. Our South Okanagan population had similar annual productivity (0.68±0.12 female fledglings per nest) to that of two Idaho populations (0.69) in similar habitat [14], all of which were much lower than an Idaho population in aspen woodland (1.0–1.2±0.5) and a South Dakota population (1.54±0.18) in post-burn forest [23]. The high productivity in the latter two populations may reflect low predator densities coupled with abundant cavities [19], [23].

### Why are habitat variables that influence nest-site selection not good at predicting nest success?

Our study is the second to report that the habitat features that predict nest-site selection for Lewis's woodpeckers are poor predictors of nest success. The authors of the first study [19] did not attempt to explain this apparent paradox. At least in terms of our study, there are several potential explanations. First, Lewis's Woodpeckers may be enmeshed in an ecological trap. Ecological traps usually occur when species use a habitat cue to assess habitat quality, but that previously reliable cue is now misleading, often because of rapid anthropogenic change [7], [58]. However, an ecological trap implies that fitness is reduced in selected sites [59], whereas in our study nest survival was neutral with respect to the habitat features that predicted site selection.

A second explanation is that Lewis's Woodpeckers do prefer nest sites that maximize their reproductive success, but these preferences are rarely realized because of interspecific competition for nest sites [3], [5], [10], [11]. In 48% of cases in our study, Lewis's Woodpeckers shared the same nest tree with European Starlings (*Sturnus vulgaris*), Northern Flickers, and/or American Kestrels (*Falco sparverius*). In several cases, we observed aggressive interactions with and nest usurpation by starlings; Lewis's Woodpeckers tended to initiate breeding later than starlings, possibly to reduce this conflict [60]. Some studies also present evidence from interior British Columbia that cavity nesters are often limited by the density of suitable cavities [33]. Thus the loss of trees with decay which are generally limiting in this area and the presence of more competitive secondary cavity nesters might result in compression of the nest web, whereby Lewis's Woodpeckers end up with sub-optimal nest sites [31]. Interestingly, in other areas, Lewis's Woodpeckers have been noted to outcompete other species for nest cavities and even usurp cavities [21], [23].

A third explanation is that nest success is limited, in part or completely, by ecological factors that are uncorrelated to habitat. Indeed, we found nest success to be primarily determined by clutch initiation date and cavity shape (cavity shape was uncorrelated with the habitat around the nest tree). As discussed earlier, both clutch initiation date and cavity shape may affect the likelihood of predation, and clutch initiation date may also be a stand-in for bird quality. Other studies have also found time-related factors to be more important than habitat features in predicting nest success [61], [62]. Together this and the previous hypothesis suggest that ecological limitations on either nest-site selection (e.g. interspecific competition) or nest success (e.g. temporal patterns in predation) mean that selected habitat features have at best weak effects on nest success.

In addition to the above explanations that address ultimate causes for why habitat features predicting nest-site selection were not correlated with nest success, a fourth suite of explanations suggests that methodological limitations prevented a real correlation from being detected. For example, our ability to detect nest-site selection effects on nest survival depends on some birds utilizing suboptimal nest sites. If nest-site selection is sufficiently strong, if optimal sites are not limited, or if population size has declined, there may be no nests in the poorest quality habitat to monitor for variation in survival, restricting the range of habitat variables we can evaluate [63], [64]. This may have occurred for three habitat variables - live tree canopy cover, nest tree decay class, and density of suitable cavities – for which selection was so extreme, and universal, that >95% of nests occurred at the 5% of available sites that maximized these variables (Table 2; compare distribution of variables in selected vs. random sites). We may also have excluded important habitat features in our models, especially given that we structured our models to include variables thought to be important in nest-site selection based on previous studies for the species elsewhere. For example, none of our random trees contained cavities, so cavity shape could not be assessed in nest-site selection models. We could not include coarse habitat features at landscape scales, including some known to affect nest survival of Lewis's Woodpeckers such as forest type (cottonwood vs. ponderosa pine: [14] and stage (recently burned vs. unburned). Finally, correlations between nest-site selection and nest success may only emerge in longer-term data sets [65].

Our study adds to the growing list of studies that report mismatches between nest-site selection and reproductive success [5], [10], [19], [43], [58], [63], [64], [66], [67], [68], [69]. However, some studies have found that habitat features that predict nest-site selection also predict nest success [3], [4], [11], [70], [71]. Therefore, there is at best only partial support in the literature for the paradigm that birds select nest sites in habitats that optimize reproductive success, especially considering that a higher proportion of the studies that observe such mismatches will remain unreported. When this paradigm is not supported, it is often because ecological factors such as interspecific competition for nest sites or temporal effects obscure or override the effects of selected habitat on nest success [3]. For example, in northern British Columbia, red squirrel nest depredation decreased during years of abundant tree mast, resulting in greatly improved chestnut-backed chickadee (*Poecile rufescens*) nest survival rates between years despite stable squirrel densities (20% vs 80%) [67].

Such mismatches between nest-site selection and nest success have important management implications. Until the reasons for these apparent paradoxes are resolved, a precautionary approach is to manage not only for preferred habitat but also to consider community wide processes such as potential interactions with predators or nest-site competitors. We recommend such an approach for Lewis's Woodpecker.

## Supporting Information

Table S1 **Contribution of each study area and year to estimates of nest-site selection and nest success in Lewis's Woodpecker, in terms of number of random sites at which vegetation was recorded, number of nests found, fate of each nest, and Mayfield estimate of nest success (mean ± S.E.).** All the study areas were mosaics of forest and grassland. The dominant forest vegetation at each site is abbreviated as OPP = Open ponderosa pine; DF = Douglas fir; RPP = riparian ponderosa pine; BCW = black cottonwood.
(DOCX)

Table S2 **Correlation coefficient matrix for the seven explanatory variables (n = 73, including 45 nesting sites and 28 random sites).** None of the correlation coefficients exceeded 0.8, the level that may result in a collinearity issue in a regression model. The associated P-value is shown in parentheses. Habitat variables include EL = elevation; TC = live tree canopy cover (%); SC = shrub cover (%); GC = grass cover (%); BA = total basal area of large trees (m^2^ per ha); ND = nest tree decay class; DS = density of suitable cavities (per ha).
(DOCX)

Table S3 **Full ranking of habitat-based models that predict Lewis's Woodpecker's nest-site selection (** ***n*** **= 73).** This is an expanded version of Table 3 in the main text. K = number of parameters in the model; −2log(L) = maximum likelihood of the model using natural logarithms; AIC_c_ = Akaike's Information Criterion for small samples; ΔAIC_c_ = adjusted AIC_c_ relative to the top model; W_i_ = AIC_c_ weight; EL = elevation; TC = live tree canopy cover; SC = shrub cover; GC = grass cover; BA = total basal area of large trees; ND = nest tree decay class; DS = density of suitable cavities.
(DOCX)

Table S4 **Full ranking of cavity and time-based models that predict Lewis's Woodpecker's daily nest survival.** This is an expanded version of Table 4 in the main text. C = clutch initiation date; NA = nest age; T = time of season; DE = ratio of hole depth to entrance size; CH = cavity height; other abbreviations as in Table S3.
(DOCX)

Table S5 **Full model ranking for habitat-based models that predict Lewis's Woodpecker daily nest survival analysis.** Global and candidate models are identical to those used to predict nest site selection (Table 3 in main text). Abbreviations are described in Table S3.
(DOCX)

Table S6 **Full model ranking for Lewis's Woodpecker daily nest survival analysis when the global and candidate models listed in Tables S4 and S6 are combined in a single model-testing framework.** Models in bold are the cavity/temporal variable models listed in Table S4. C = clutch initiation date; NA = nest age; T = time of season; DE = ratio of hole depth to entrance size; CH = cavity height; other abbreviations as in Table S3.
(DOCX)
