# Supplementary material for: Habitat Selection and Reproductive Success of Lewis's Woodpecker (Melanerpes lewis) at Its Northern Limit
Source: PLoS One. 2012 Sep 18;7(9):e44346. doi: 10.1371/journal.pone.0044346 (PMC3445559; doi:10.1371/journal.pone.0044346)
Supplement: Table S1 — Contribution of each study area and year to estimates of nest-site selection and nest success in Lewis's Woodpecker, in terms of number of random sites at which vegetation was recorded, number of nests found, fate of each nest, and Mayfield estimate of nest success (mean ± S.E.). All the study areas were mosaics of forest and grassland. The dominant forest vegetation at each site is abbreviated as OPP = Open ponderosa pine; DF = Douglas fir; RPP = riparian ponderosa pine; BCW = black cottonwood. (DOCX) [file pone.0044346.s001.docx]

| Study Area | Dominant | Number | 2004 | 2005 | Pooled | Nest success |
| --- | --- | --- | --- | --- | --- | --- |
|  | Vegetation | of random | Failed/ | Failed/ | Failed/ | (Pooled |
|  |  | Sites | Total | Total | Total | data) |
| Chopaka | OPP | 4 | 0/1 | 4/4 | 4/5 | 0.12±0.13 |
| Kilpoola | OPP+DF | 7 | 4/7 | 2/9 | 6/16 | 0.49±0.14 |
| Spotted Lake | OPP | 4 | 2/3 | 0/2 | 2/5 | 0.47±0.25 |
| SunOka | RPP+BCW | 1 | 0/2 | 1/3 | 1/5 | 0.71±0.24 |
| Vaseux Lake | OPP+DF+RPP | 8 | 2/4 | 3/9 | 5/13 | 0.49±0.16 |
| Surrounding areas | OPP+RPP+DF | 4 | 0/4 | 2/9 | 2/13 | 0.77±0.14 |
