# Supplementary material for: Habitat Selection and Reproductive Success of Lewis's Woodpecker (Melanerpes lewis) at Its Northern Limit
Source: PLoS One. 2012 Sep 18;7(9):e44346. doi: 10.1371/journal.pone.0044346 (PMC3445559; doi:10.1371/journal.pone.0044346)
Supplement: Table S2 — Correlation coefficient matrix for the seven explanatory variables (n = 73, including 45 nesting sites and 28 random sites). None of the correlation coefficients exceeded 0.8, the level that may result in a collinearity issue in a regression model. The associated P-value is shown in parentheses. Habitat variables include EL = elevation; TC = live tree canopy cover (%); SC = shrub cover (%); GC = grass cover (%); BA = total basal area of large trees (m2 per ha); ND = nest tree decay class; DS = density of suitable cavities (per ha). (DOCX) [file pone.0044346.s002.docx]

|  | EL | TC | SC | GC | BA | ND |
| --- | --- | --- | --- | --- | --- | --- |
| TC | -0.2406 |  |  |  |  |  |
|  | (0.0404) |  |  |  |  |  |
| SC | 0.1324 | 0.0429 |  |  |  |  |
|  | (0.2642) | (0.7183) |  |  |  |  |
| GC | 0.1651 | -0.5049 | -0.0299 |  |  |  |
|  | (0.1628) | (0.000) | (0.802) |  |  |  |
| BA | 0.3677 | 0.1609 | 0.0115 | 0.142 |  |  |
|  | (0.0014) | (0.1738) | (0.9233) | (0.2308) |  |  |
| ND | 0.5669 | -0.5261 | 0.1682 | 0.2534 | 0.2178 |  |
|  | (0.000) | (0.000) | (0.1549) | (0.0305) | (0.0641) |  |
| DS | 0.1421 | -0.2339 | -0.1128 | 0.3909 | 0.3768 | 0.3454 |
|  | (0.2303) | (0.0465) | (0.342) | (0.0006) | (0.001) | (0.0028) |
