# Supplementary material for: Habitat Selection and Reproductive Success of Lewis's Woodpecker (Melanerpes lewis) at Its Northern Limit
Source: PLoS One. 2012 Sep 18;7(9):e44346. doi: 10.1371/journal.pone.0044346 (PMC3445559; doi:10.1371/journal.pone.0044346)
Supplement: Table S3 — Full ranking of habitat-based models that predict Lewis's Woodpecker's nest-site selection ( n = 73). This is an expanded version of Table 3 in the main text. K = number of parameters in the model; −2log(L) = maximum likelihood of the model using natural logarithms; AICc = Akaike's Information Criterion for small samples; ΔAICc = adjusted AICc relative to the top model; Wi = AICc weight; EL = elevation; TC = live tree canopy cover; SC = shrub cover; GC = grass cover; BA = total basal area of large trees; ND = nest tree decay class; DS = density of suitable cavities. (DOCX) [file pone.0044346.s003.docx]

| **Model** | **K** | **-2log(L)** | **AICc** | **∆AICc** | **Wi** |
| --- | --- | --- | --- | --- | --- |
| EL+TC+BA+ND+DS | 6 | 30.23 | 43.50 | 0.00 | 0.49 |
| EL+TC+BA+ND+DS+SC | 7 | 29.59 | 45.31 | 1.81 | 0.20 |
| EL+TC+BA+ND+DS+GC | 7 | 30.20 | 45.92 | 2.42 | 0.14 |
| EL+TC+BA+ND+DS+SC+GC | 8 | 29.59 | 47.84 | 4.33 | 0.06 |
| EL+BA+ND+DS+SC+GC | 7 | 32.30 | 48.03 | 4.52 | 0.05 |
| EL+TC+SC+GC+ND+DS | 7 | 34.13 | 49.85 | 6.35 | 0.02 |
| TC+BA+ND+DS | 5 | 40.41 | 51.31 | 7.81 | 0.01 |
| GC+BA+ND+DS | 5 | 41.24 | 52.14 | 8.63 | 0.01 |
| TC+GC+ND+DS | 5 | 41.59 | 52.49 | 8.98 | 0.01 |
| BA+ND+DS | 4 | 44.16 | 52.75 | 9.24 | 0.00 |
| TC+GC+BA+ND+DS | 6 | 39.60 | 52.88 | 9.37 | 0.00 |
| TC+SC+GC+BA+ND+DS | 7 | 37.57 | 53.30 | 9.79 | 0.00 |
| TC+GC+BA+DS | 5 | 42.61 | 53.50 | 10.00 | 0.00 |
| EL+TC+SC+GC+BA+DS | 7 | 37.85 | 53.58 | 10.07 | 0.00 |
| DS | 2 | 50.57 | 54.74 | 11.24 | 0.00 |
| EL+TC+SC+GC+BA+ND | 7 | 47.83 | 63.56 | 20.05 | 0.00 |
| ND | 2 | 82.27 | 86.44 | 42.93 | 0.00 |
| EL+TC+SC+GC | 5 | 81.33 | 92.22 | 48.72 | 0.00 |
| TC | 2 | 88.74 | 92.91 | 49.41 | 0.00 |
| GC | 2 | 89.75 | 93.92 | 50.42 | 0.00 |
| BA | 2 | 91.41 | 95.58 | 52.08 | 0.00 |
| SC | 2 | 94.93 | 99.10 | 55.60 | 0.00 |
| Constant | 1 | 97.20 | 99.26 | 55.76 | 0.00 |
| EL | 2 | 96.44 | 100.61 | 57.11 | 0.00 |
