# Supplementary material for: Habitat Selection and Reproductive Success of Lewis's Woodpecker (Melanerpes lewis) at Its Northern Limit
Source: PLoS One. 2012 Sep 18;7(9):e44346. doi: 10.1371/journal.pone.0044346 (PMC3445559; doi:10.1371/journal.pone.0044346)
Supplement: Table S4 — Full ranking of cavity and time-based models that predict Lewis's Woodpecker's daily nest survival. This is an expanded version of Table 4 in the main text. C = clutch initiation date; NA = nest age; T = time of season; DE = ratio of hole depth to entrance size; CH = cavity height; other abbreviations as in Table S3. (DOCX) [file pone.0044346.s004.docx]

| **Model** | **K** | **-2log(L)** | **AICc** | **∆AICc** | **Wi** |
| --- | --- | --- | --- | --- | --- |
| DE+c+c^2^+NA+NA^2^ | 6 | 144.35 | 156.42 | 0.00 | 0.42 |
| Ch+c+c^2^+NA+NA^2^ | 6 | 146.18 | 158.25 | 1.83 | 0.17 |
| DE+NA+NA^2^ | 4 | 150.69 | 158.72 | 2.30 | 0.13 |
| c+c^2^+NA+NA^2^ | 5 | 149.04 | 159.09 | 2.67 | 0.11 |
| Ch+NA+NA^2^ | 4 | 153.53 | 161.56 | 5.14 | 0.03 |
| DE+Ch+c+c^2^+NA+NA^2^+t+t^2^+year | 10 | 141.70 | 161.88 | 5.46 | 0.03 |
| DE+t+t^2^+NA+NA^2^ | 6 | 149.82 | 161.89 | 5.47 | 0.03 |
| NA+NA^2^ | 3 | 156.38 | 164.41 | 7.99 | 0.02 |
| DE+c+c^2^+NA | 5 | 152.53 | 162.58 | 6.16 | 0.02 |
| DE+c+c^2^ | 4 | 155.06 | 163.09 | 6.67 | 0.01 |
| DE+Ch+c+c^2^ | 5 | 154.13 | 164.18 | 7.75 | 0.01 |
| Ch+c+c^2^ | 4 | 156.67 | 164.70 | 8.28 | 0.01 |
| c+c^2^ | 3 | 159.23 | 165.25 | 8.83 | 0.01 |
| t+t^2^+NA+NA^2^ | 5 | 155.91 | 165.96 | 9.54 | 0.00 |
| Constant | 1 | 169.27 | 171.27 | 14.85 | 0.00 |
| t+t^2^ | 3 | 166.69 | 172.71 | 16.28 | 0.00 |
| year | 2 | 169.05 | 173.06 | 16.63 | 0.00 |
