# Supplementary material for: Habitat Selection and Reproductive Success of Lewis's Woodpecker (Melanerpes lewis) at Its Northern Limit
Source: PLoS One. 2012 Sep 18;7(9):e44346. doi: 10.1371/journal.pone.0044346 (PMC3445559; doi:10.1371/journal.pone.0044346)
Supplement: Table S5 — Full model ranking for habitat-based models that predict Lewis's Woodpecker daily nest survival analysis. Global and candidate models are identical to those used to predict nest site selection (Table 3 in main text). Abbreviations are described in Table S3. (DOCX) [file pone.0044346.s005.docx]

| **Model** | **n** | **K** | **-2log(L)** | **AICc** | **∆AICc** | **Wi** |
| --- | --- | --- | --- | --- | --- | --- |
| GC | 1251 | 2 | 167.12 | 171.13 | 0.00 | 0.26 |
| EL+TC+SC+GC | 1251 | 5 | 163.00 | 173.05 | 1.92 | 0.10 |
| CONSTANT | 1251 | 1 | 171.29 | 173.29 | 2.16 | 0.09 |
| ND | 1251 | 2 | 170.23 | 174.24 | 3.11 | 0.06 |
| TC+GC+ND+DS | 1251 | 5 | 164.47 | 174.52 | 3.39 | 0.05 |
| GC+BA+ND+DS | 1251 | 5 | 164.49 | 174.54 | 3.41 | 0.05 |
| DS | 1251 | 2 | 170.53 | 174.54 | 3.41 | 0.05 |
| EL | 1251 | 2 | 170.69 | 174.70 | 3.58 | 0.04 |
| SC | 1251 | 2 | 171.13 | 175.14 | 4.01 | 0.04 |
| BA | 1251 | 2 | 171.13 | 175.14 | 4.02 | 0.04 |
| TC | 1251 | 2 | 171.28 | 175.29 | 4.16 | 0.03 |
| TC+GC+BA+DS | 1251 | 5 | 165.81 | 175.86 | 4.73 | 0.02 |
| BA+ND+DS | 1251 | 4 | 168.06 | 176.09 | 4.96 | 0.02 |
| TC+GC+BA+ND+DS | 1251 | 6 | 164.45 | 176.52 | 5.39 | 0.02 |
| EL+TC+SC+GC+BA+ND | 1251 | 7 | 162.53 | 176.62 | 5.49 | 0.02 |
| EL+BA+ND+DS+SC+GC | 1251 | 7 | 162.61 | 176.70 | 5.57 | 0.02 |
| EL+TC+SC+GC+ND+DS | 1251 | 7 | 162.67 | 176.76 | 5.63 | 0.02 |
| EL+TC+SC+GC+BA+DS | 1251 | 7 | 162.79 | 176.88 | 5.75 | 0.01 |
| EL+TC+BA+ND+DS+GC | 1251 | 7 | 162.90 | 177.00 | 5.87 | 0.01 |
| TC+SC+GC+BA+ND+DS | 1251 | 7 | 163.61 | 177.70 | 6.57 | 0.01 |
| TC+BA+ND+DS | 1251 | 5 | 167.72 | 177.77 | 6.64 | 0.01 |
| EL+TC+BA+ND+DS+SC+GC | 1251 | 8 | 162.46 | 178.58 | 7.45 | 0.01 |
| EL+TC+BA+ND+DS | 1251 | 6 | 166.72 | 178.78 | 7.65 | 0.01 |
| EL+TC+BA+ND+DS+SC | 1251 | 7 | 166.65 | 180.74 | 9.62 | 0.00 |
