# Supplementary material for: Habitat Selection and Reproductive Success of Lewis's Woodpecker (Melanerpes lewis) at Its Northern Limit
Source: PLoS One. 2012 Sep 18;7(9):e44346. doi: 10.1371/journal.pone.0044346 (PMC3445559; doi:10.1371/journal.pone.0044346)
Supplement: Table S6 — Full model ranking for Lewis's Woodpecker daily nest survival analysis when the global and candidate models listed in Tables S4 and S6 are combined in a single model-testing framework. Models in bold are the cavity/temporal variable models listed in Table S4. C = clutch initiation date; NA = nest age; T = time of season; DE = ratio of hole depth to entrance size; CH = cavity height; other abbreviations as in Table S3. (DOCX) [file pone.0044346.s006.docx]

| **Model** | **n** | **K** | **-2log(L)** | **AICc** | **∆AICc** | **Wi** |
| --- | --- | --- | --- | --- | --- | --- |
| **DE+C+C^2^+NA+NA^2^** | **1251** | **6** | **144.35** | **156.42** | **0.00** | **0.42** |
| **NH+C+C^2^+NA+NA^2^** | **1251** | **6** | **146.18** | **158.25** | **1.83** | **0.17** |
| **DE+NA+NA^2^** | **1251** | **4** | **150.69** | **158.72** | **2.30** | **0.13** |
| **C+C^2^+NA+NA^2^** | **1251** | **5** | **149.04** | **159.09** | **2.67** | **0.11** |
| **NH+NA+NA^2^** | **1251** | **4** | **153.79** | **161.82** | **5.40** | **0.03** |
| **DE+NH+C+C^2^+NA+NA^2^+T+ T^2^+YEAR** | **1251** | **10** | **141.70** | **161.88** | **5.46** | **0.03** |
| **DE+T+T^2^+NA+NA^2^** | **1251** | **6** | **149.82** | **161.89** | **5.47** | **0.03** |
| **NA+NA^2^** | **1251** | **3** | **156.55** | **162.57** | **6.14** | **0.02** |
| **DE+C+C^2^+NA** | **1251** | **5** | **152.53** | **162.58** | **6.16** | **0.02** |
| **DE+C+C^2^** | **1251** | **4** | **155.06** | **163.09** | **6.67** | **0.02** |
| **DE+NH+C+C^2^** | **1251** | **5** | **154.16** | **164.20** | **7.78** | **0.01** |
| **NH+C+C^2^** | **1251** | **4** | **157.26** | **165.29** | **8.87** | **0.01** |
| **T+T^2^+NA+NA^2^** | **1251** | **5** | **155.91** | **165.96** | **9.54** | **0.00** |
| **C+C^2^** | **1251** | **3** | **160.05** | **166.07** | **9.65** | **0.00** |
| GC | 1251 | 2 | 167.12 | 171.13 | 14.71 | 0.00 |
| EL+TC+SC+GC | 1251 | 5 | 163.00 | 173.05 | 16.63 | 0.00 |
| CONSTANT | 1251 | 1 | 171.29 | 173.29 | 16.87 | 0.00 |
| **T+T^2^** | **1251** | **3** | **167.35** | **173.37** | **16.95** | **0.00** |
| ND | 1251 | 2 | 170.23 | 174.24 | 17.82 | 0.00 |
| TC+GC+ND+DS | 1251 | 5 | 164.47 | 174.52 | 18.10 | 0.00 |
| GC+BA+ND+DS | 1251 | 5 | 164.49 | 174.54 | 18.11 | 0.00 |
| DS | 1251 | 2 | 170.53 | 174.54 | 18.12 | 0.00 |
| EL | 1251 | 2 | 170.69 | 174.70 | 18.28 | 0.00 |
| YEAR | **1251** | **2** | **171.03** | **175.04** | **18.62** | **0.00** |
| SC | 1251 | 2 | 171.13 | 175.14 | 18.72 | 0.00 |
| BA | 1251 | 2 | 171.13 | 175.14 | 18.72 | 0.00 |
| TC | 1251 | 2 | 171.28 | 175.29 | 18.87 | 0.00 |
| TC+GC+BA+DS | 1251 | 5 | 165.81 | 175.86 | 19.44 | 0.00 |
| BA+ND+DS | 1251 | 4 | 168.06 | 176.09 | 19.67 | 0.00 |
| BA+ND+DS | 1251 | 4 | 168.06 | 176.09 | 19.67 | 0.00 |
| TC+GC+BA+ND+DS | 1251 | 6 | 164.45 | 176.52 | 20.10 | 0.00 |
| EL+TC+SC+GC+BA+ND | 1251 | 7 | 162.53 | 176.62 | 20.20 | 0.00 |
| EL+BA+ND+DS+SC+GC | 1251 | 7 | 162.61 | 176.70 | 20.28 | 0.00 |
| EL+TC+SC+GC+ND+DS | 1251 | 7 | 162.67 | 176.76 | 20.34 | 0.00 |
| EL+TC+SC+GC+BA+DS | 1251 | 7 | 162.79 | 176.88 | 20.46 | 0.00 |
| EL+TC+GC+BA+ND+DS | 1251 | 7 | 162.90 | 177.00 | 20.57 | 0.00 |
| TC+SC+GC+BA+ND+DS | 1251 | 7 | 163.61 | 177.70 | 21.28 | 0.00 |
| TC+BA+ND+DS | 1251 | 5 | 167.72 | 177.77 | 21.35 | 0.00 |
| EL+TC+SC+GC+BA+ND+DS | 1251 | 8 | 162.46 | 178.58 | 22.16 | 0.00 |
| EL+TC+BA+ND+DS | 1251 | 6 | 166.72 | 178.78 | 22.36 | 0.00 |
| EL+TC+SC+BA+ND+DS | 1251 | 7 | 166.65 | 180.74 | 24.32 | 0.00 |
